# Management of Persistent Pruritus and Lichenoid Reaction Secondary to Nivolumab With Narrowband Ultraviolet B Phototherapy

**DOI:** 10.3389/fonc.2018.00405

**Published:** 2018-09-25

**Authors:** Marie Donaldson, Joshua L. Owen, Young K. Chae, Jennifer N. Choi

**Affiliations:** ^1^Department of Dermatology, Northwestern University Feinberg School of Medicine, Chicago, IL, United States; ^2^Robert H. Lurie Comprehensive Cancer Center of Northwestern University, Chicago, IL, United States; ^3^Department of Medicine, Northwestern University Feinberg School of Medicine, Chicago, IL, United States

**Keywords:** PD-1 inhibitor, nivolumab, lichenoid, dermatitis, immunotherapy, phototherapy, non-small cell lung cancer

## Abstract

Immune checkpoint inhibitors targeting the programmed cell death receptor 1 (PD-1) are increasingly used to treat several malignancies, with the most common adverse event being cutaneous toxicity. We report the case of a 68-years-old man with stage IV non-small cell lung cancer treated with nivolumab who developed a pruritic, lichenoid eruption refractory to treatment with topical or systemic steroids, who was started on narrow band ultraviolet B therapy which resolved the reaction.

## Background

Immune checkpoint inhibitors targeting the programmed cell death receptor 1 (PD-1) are increasingly used to treat several malignancies, including non-small cell lung cancer (NSCLC), metastatic melanoma, and urogenital cancers (1). The most common adverse event (AE) from this class of medication is cutaneous toxicity, occurring in up to 49% of patients treated with PD-1 inhibitors (2). Common cutaneous AEs include pruritus, rash, and vitiligo (3). The mechanism for these eruptions is not fully understood, though it involves CD4^+^- and CD8^+^-T cells likely activated by the immunotherapy leading to the targeting of an antigen in the epidermis or dermis (4, 5). Cutaneous AEs rarely require cessation of therapy, and their development may be an indicator of increased response to treatment (3).One type of mucocutaneous reaction that has been described with the use of PD-1 inhibitors is a lichenoid dermatitis (3, 6). The lichenoid reaction to anti-PD-1 therapy has been shown to have more spongiosis and epidermal necrosis on histopathology when compared to other lichenoid reactions (4). Treatment for these eruptions often consists of skin-directed therapy with topical emollients, topical corticosteroids (4), and oral antipruritics, such as antihistamines (7). For severe (grade 3–4) reactions, systemic glucocorticoid administration and delay or discontinuation of anti-PD-1 therapy should be considered.

Narrowband ultraviolet B (NBUVB) phototherapy is another skin-directed treatment modality used for a variety of conditions in dermatology, including lichenoid disorders like lichen planus (7).We present the case of a patient with stage IV NSCLC who developed a persistent and pruritic lichenoid eruption while on treatment with nivolumab that was refractory to topical and systemic glucocorticoids, but resolved with NBUVB phototherapy. Written informed consent was obtained from the patient for the publication of this manuscript.

## Case report

**Figure 1:**
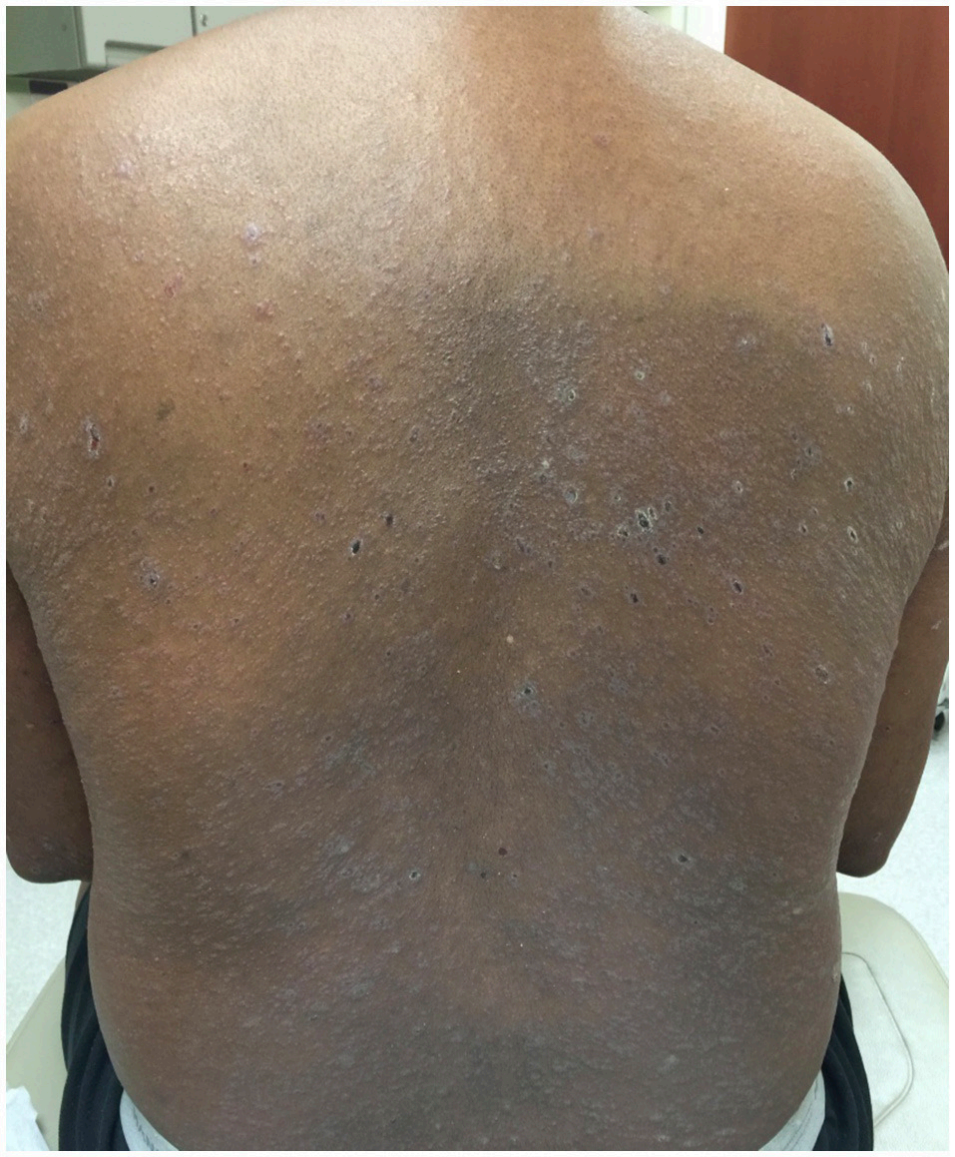
Clinical image of the cutaneous eruption that developed after six cycles of nivolumab. On the back, there were widespread and numerous 3–10 mm pink to pink-brown thin flat-topped papules and plaques with scale.

**Figure 2:**
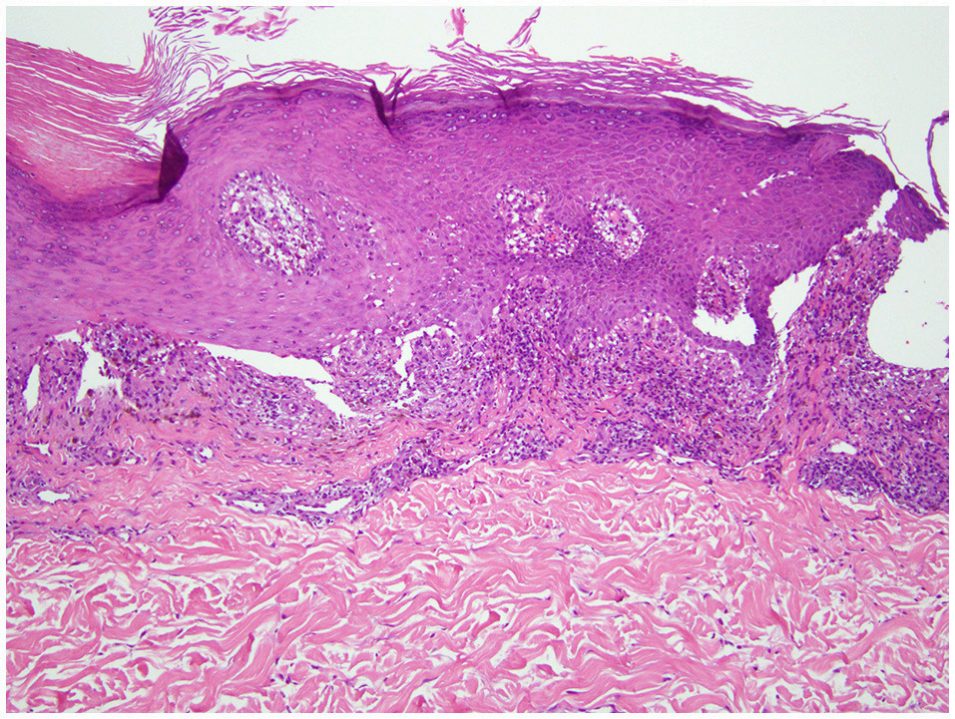
Punch biopsy of a representative lesion (left upper arm). Hematoxylin and eosin staining revealed an acanthotic epidermis with hyperkeratosis and hypergranulosis. There is a band-like lymphohistiocytic infiltrate at the dermal-epidermal junction with focal squamatization of the basal cell layer and scattered necrotic keratinocytes (20 × magnification).

**Figure 3:**
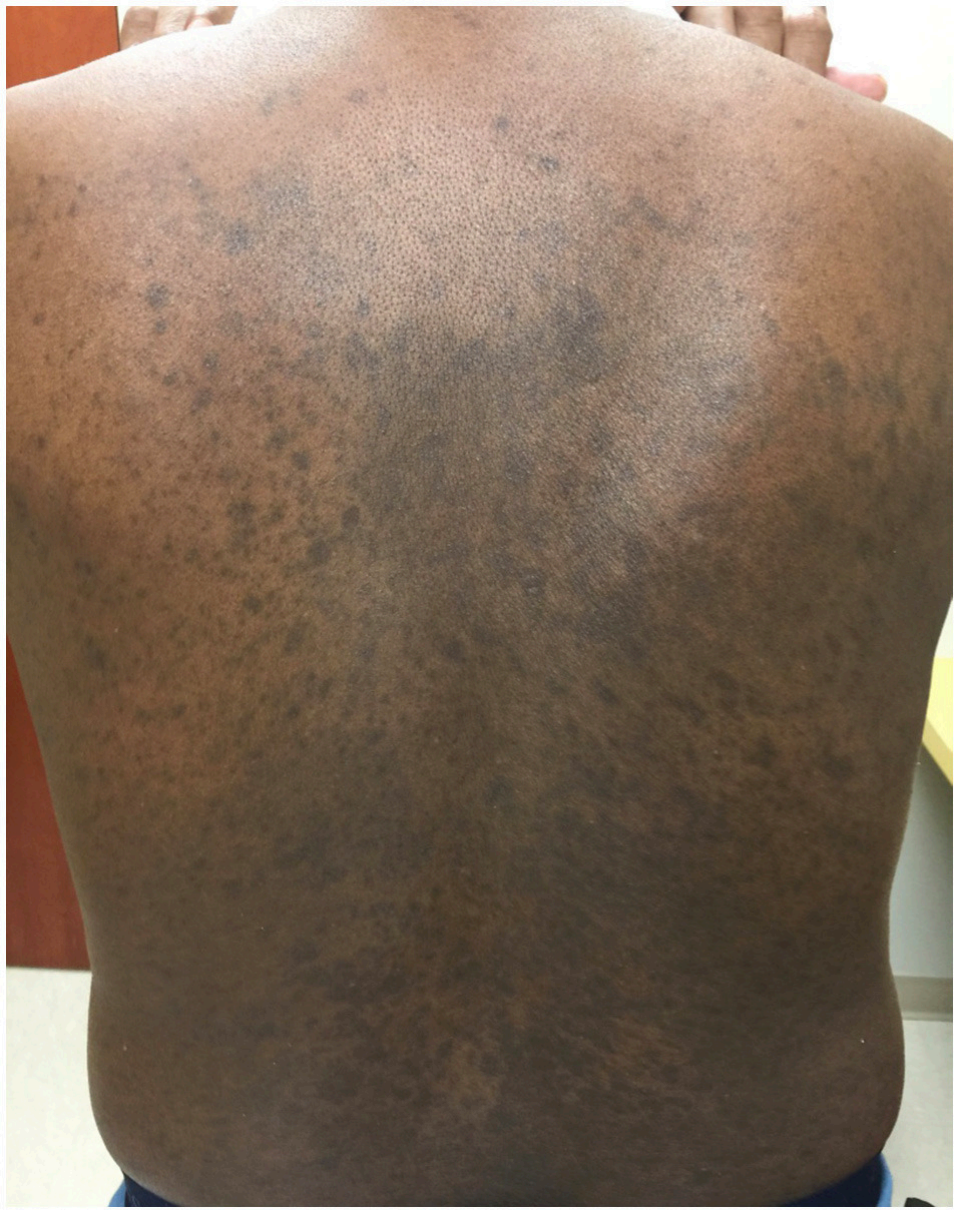
Clinical image after successful treatment with NBUVB. On the back, there were widespread and numerous hyperpigmented macules coalescing into patches. There was no erythema or scale.

A 68-years-old man, with a history of diffuse lichen planus which had resolved 9 years prior, was diagnosed with stage IV squamous NSCLC. He underwent definitive radiation therapy to the right upper lung lobe with carboplatin and paclitaxel combination chemotherapy weekly for 6 weeks, with positive response to therapy. However, after 9 months, his lung nodules were noted to be progressively enlarging, and two additional nodules were identified, concerning for new metastases. He was then started on nivolumab, a PD-1 inhibitor, at a dose of 3 mg/kg infused every 2 weeks. After six cycles of treatment, he developed a widespread pruritic eruption involving chest, back, extremities, and penis. On examination, he was noted to have too numerous to count 3–10 mm pink to pink-brown thin papules and plaques, which were flat-topped with scale over the chest, abdomen, back (Figure 1), arms, legs, and penile shaft, some of which had an erythematous base. The head of the penis had numerous ill-defined erosions measuring up to 1.5 cm. Additionally, he had developed a 5 mm shallow ulceration of the left lateral tongue. A punch biopsy of a characteristic lesion on the left upper arm was performed, which showed a slightly acanthotic epidermis with prominent hyperkeratosis and hypergranulosis, with a band-like lymphohistiocytic infiltrate, focal squamatization of the basal cell layer, and necrotic keratinocytes (Figure 2). Given the clinical presentation and these histopathological changes, he was diagnosed with a lichenoid mucocutaneous eruption due to nivolumab. Treatment with triamcinolone 0.1% ointment to the body, clobetasol 0.05% ointment to the penis, and clobetasol 0.05% gel to the tongue twice daily was initiated. Given the widespread distribution of the eruption and the associated intense pruritus, a 5-weeks oral prednisone taper starting at 80 mg daily was also started and nivolumab treatment was held for 1 week. At the completion of the oral steroid taper, his rash had significantly improved, including complete resolution of the penile erosions and oral ulceration despite resuming nivolumab therapy. However, 6 days after discontinuing prednisone, the rash recurred on the chest and back, requiring a second prednisone taper. The eruption recurred again after completing the taper, leading to a trial 5 mg daily maintenance dose of prednisone. Due to persistence of rash and pruritus, a third prednisone taper was initiated and narrowband UVB phototherapy three times per week was started as an adjunct therapy. The prednisone was gradually transitioned to a 10 mg daily maintenance dosing. After 1 month of this treatment regimen, the patient's eruption had significantly improved, with no inflammatory papules seen on examination. Initially the prednisone maintenance dose was unable to be decreased without recurrence of the eruption. After ~70 NBUVB treatments (5–6 months), a slow prednisone taper with oral hydroxyzine was initiated. Once on 5 mg daily prednisone, the patient elected to stop NBUVB treatments as his rash and pruritus were controlled. On clinical examination 6 months later, complete resolution of the rash was observed with only residual post-inflammatory hyperpigmentation while still on 4 mg daily prednisone (Figure 3). His prednisone continued to be tapered, eventually being discontinued completely after 4 weeks. Four months after stopping prednisone, the rash and pruritus have remained completely resolved. Importantly, while the patient has been treated for this rash, he has been able to remain on nivolumab therapy. To date, he has completed 52 cycles and his NSCLC has been stable.

## Discussion

The use of immune checkpoint inhibitors for the treatment of a growing list of malignancies has led to a greater clinical appreciation of the range of cutaneous AEs. As these are the most commonly encountered AEs with this class of medication, it is essential for physicians caring for such patients to have an understanding of both skin-directed and systemic therapies to treat these eruptions so that patients can remain on treatment. Nivolumab has been shown to have an overall response rate of 30% (1), and it can be an essential element to the management and prognosis of patients with NSCLC. Depending on the severity of the eruption, the cutaneous AEs of the drug threaten patient adherence to treatment. For grade 1–2 reactions, skin-directed therapy is often sufficient; however, for grade 3–4 reactions systemic glucocorticoids are often required to help control the eruption. Systemic steroids have their own attendant side effects, including adrenal insufficiency, diabetes mellitus, weight gain, and osteoporosis.

This report details a patient who developed a severe lichenoid dermatitis during nivolumab treatment, requiring postponement of therapy until systemic steroid treatment was able to control the rash. Unfortunately, the eruption remained steroid-dependent despite numerous attempts at steroid taper. By introducing NBUVB phototherapy to his skin-directed regimen, the dose of systemic steroids was able to be decreased and eventually stopped completely, while allowing the patient to remain on immunotherapy treatment.

The mechanism of NBUVB in treatment of an array of dermatoses is not fully understood; some of the purported mechanisms include altering cytokines, causing DNA damage, decreasing proliferation, inducing apoptosis, and promoting immunosuppression (8, 9). Treatment of the lichenoid eruption due to anti-PD-1 with a skin-directed anti-inflammatory therapy like NBUVB therapy is reasonable given the involvement of both CD4^+^- and CD8^+^-T cells in the process. Other lichenoid disorders have also been successfully treated with NBUVB (9).

The treatment of the cutaneous AEs related to immune checkpoint inhibitors will continue to be a growing area of clinical need. Discovering new and creative ways to address these toxicities will improve adherence and tolerability of these therapies. As is demonstrated in this case report, NBUVB is a viable treatment option for the treatment of patients with a lichenoid eruption due to anti-PD-1 therapy. This may reduce long-term systemic steroid use. Further study is warranted to look at the efficacy of NBUVB for immunotherapy-induced rash in a larger cohort of patients.

## Author contributions

MD, JO, YC, and JC contributed to drafting, editing, and reviewing of the manuscript. JC and YC developed the idea for the paper.

### Conflict of interest statement

The authors declare that the research was conducted in the absence of any commercial or financial relationships that could be construed as a potential conflict of interest.

## References

[1] Sunshine J Taube JM. PD-1/PD-L1 inhibitors. Curr Opin Pharmacol. (2015) 23:32–8. 10.1016/j.coph.2015.05.011 26047524 PMC4516625

[2] Hwang SJ Carlos G Wakade D Byth K Kong BY Chou S. Cutaneous adverse events (AEs) of anti-programmed cell death (PD)-1 therapy in patients with metastatic melanoma: a single-institution cohort. J Am Acad Dermatol. (2016) 74:455–61.e451. 10.1016/j.jaad.2015.10.029 26793994

[3] Sibaud V Meyer N Lamant L Vigarios E Mazieres J Delord JP. Dermatologic complications of anti-PD-1/PD-L1 immune checkpoint antibodies. Curr Opin Oncol. (2016) 28:254–63. 10.1097/cco.0000000000000290 27136138

[4] Schaberg KB Novoa RA Wakelee HA Kim J Cheung C Srinivas S. Immunohistochemical analysis of lichenoid reactions in patients treated with anti-PD-L1 and anti-PD-1 therapy. J Cutan Pathol. (2016) 43:339–46. 10.1111/cup.12666 26762844

[5] Khoja L Day D Wei-Wu Chen T Siu LL Hansen AR. Tumour- and class-specific patterns of immune-related adverse events of immune checkpoint inhibitors: a systematic review. Ann Oncol. (2017) 28:2377–85. 10.1093/annonc/mdx286 28945858

[6] Shi VJ Rodic N Gettinger S Leventhal JS Neckman JP Girardi M. Clinical and histologic features of lichenoid mucocutaneous eruptions due to anti-programmed cell death 1 and anti-programmed cell death ligand 1 immunotherapy. JAMA Dermatol. (2016) 152:1128–36. 10.1001/jamadermatol.2016.2226 27411054 PMC6108080

[7] Eigentler TK Hassel JC Berking C Aberle J Bachmann O Grunwald V. Diagnosis, monitoring and management of immune-related adverse drug reactions of anti-PD-1 antibody therapy. Cancer Treat Rev. (2016) 45:7–18. 10.1016/j.ctrv.2016.02.003 26922661

[8] Reich A Medrek K. Effects of narrow band UVB (311 nm) irradiation on epidermal cells. Int J Mol Sci. (2013) 14:8456–66. 10.3390/ijms14048456 23594996 PMC3645754

[9] Totonchy MB Chiu MW. UV-based therapy. Dermatol Clin. (2014). 32:399–413, ix–x. 10.1016/j.det.2014.03.003 24891061

